# Apigenin as a Promising Agent for Enhancing Female Reproductive Function and Treating Associated Disorders

**DOI:** 10.3390/biomedicines12102405

**Published:** 2024-10-21

**Authors:** Alexander V. Sirotkin, Abdel Halim Harrath

**Affiliations:** 1 Faculty of Natural Sciences, Constantine the Philosopher University in Nitra, 949 74 Nitra, Slovakia; asirotkin@ukf.sk; 2 Zoology Department, College of Science, King Saud University, P.O. Box 2455, Riyadh 11451, Saudi Arabia

**Keywords:** apigenin, antioxidants, ovary, hormone, fecundity, cancer

## Abstract

Apigenin is an organic flavonoid abundant in some plants such as parsley, chamomile, or celery. Recently, it has been investigated for several of its pharmacological characteristics, such as its ability to act as an antioxidant, reduce inflammation, and inhibit the growth of cancer cells. The purpose of this review is to provide a summary of the existing knowledge regarding the effects of apigenin on female reproductive systems and its dysfunctions. Apigenin can influence reproductive processes by regulating multiple biological events, including oxidative processes, cell proliferation, apoptosis, cell renewal and viability, ovarian blood supply, and the release of reproductive hormones. It could stimulate ovarian folliculogenesis, as well as ovarian and embryonal cell proliferation and viability, which can lead to an increase in fertility and influence the release of reproductive hormones, which may exert its effects on female reproductive health. Furthermore, apigenin could inhibit the activities of ovarian cancer cells and alleviate the pathological changes in the female reproductive system caused by environmental pollutants, harmful medications, cancer, polycystic ovarian syndrome, ischemia, as well as endometriosis. Therefore, apigenin may have potential as a biostimulator for female reproductive processes and as a therapeutic agent for certain reproductive diseases.

## 1. Introduction

Apigenin, also known as 4′,5,7-trihydroxyflavone, belongs to the flavonoid family, which consists of polyphenolic chemicals that are recognized for their antioxidant, anti-inflammatory, and anticancer characteristics [1]. Apigenin has traditionally been obtained from medicinal herbs, indicating a longstanding recognition of its beneficial properties [2]. The basic flavone skeleton is arranged into two aromatic rings linked by a heterocyclic pyran ring possessing hydroxyl groups at positions C-5 and C-7 of the ring, with pyran and C-4′ of the other aromatic ring [3]. The effect of apigenin in regulating ovarian function is very remarkable and can improve the number of eggs in the ovaries by decreasing the harmful effects of oxidative stress and cell death in ovarian cells [4]. Preserving ovarian function is essential, particularly considering the loss in female fertility associated with aging [5]. The antioxidant capabilities of apigenin aid in reducing the harm produced by reactive oxygen species (ROS), which are recognized to hinder ovarian function and expedite the aging of ovarian cells [6]. The molecular mechanism and benefits of apigenin in the female reproductive system are crucial for female health, although there is a lack of clarity regarding its precise molecular activity.

The female reproductive system has the responsibility to produce gametes, known as eggs or ova, as well as specific sex hormones [7]. It also supports the development of fertilized eggs into mature fetuses, preparing them for conception [8]. Apigenin’s diverse range of biological actions makes it a highly promising option for improving female reproductive function and addressing related diseases [9]. The anti-inflammatory, antioxidant, anticancer, hormone-modulating, and neuroprotective activities of this substance establish a strong basis for its prospective therapeutic effects [10]. Apigenin also plays a fascinating role in regulating hormone levels and enhancing ovarian function [11]. Apigenin has been demonstrated to affect the secretion of important reproductive hormones, such as estrogen and progesterone, which are commonly disrupted in many reproductive diseases [4]. Hormonal modulation can effectively restore regular menstrual cycles along with improving reproductive outcomes in women suffering disorders such as polycystic ovary syndrome (PCOS) and mysterious infertility [12]. In the current review, we provide a concise summary of apigenin’s potential as a beneficial substance for improving female reproductive function and addressing associated diseases and mechanisms of action, as well as the utilization of apigenin in the field of reproductive biology and medicine (Table S1).

## 2. Origin and Characteristics Which Determine the Physiological Effects of Apigenin

**Figure 1:**
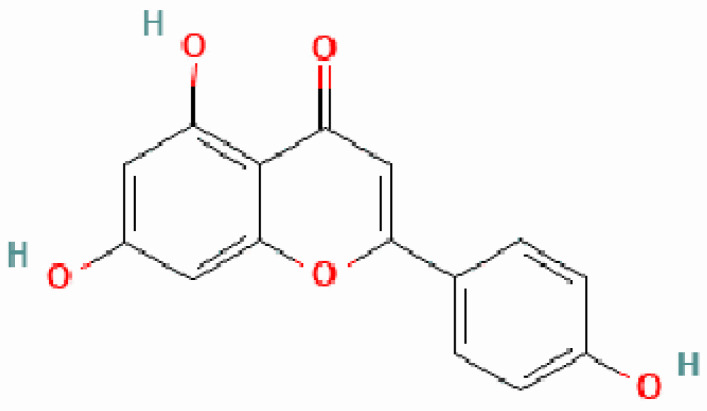
Chemical structure of apigenin (https://pubchem.ncbi.nlm.nih.gov/compound/5280443#section=2D-Structure, accessed on 12 October 2024).

Apigenin is a compound with the chemical formula C15H10O5 and the systematic name 5,7-dihydroxy-2-(4-hydroxyphenyl)chromen-4-one [13]. It is identified by the PubChem ID 5280443. The chemical structure can be found in Figure 1 at the following link: https://pubchem.ncbi.nlm.nih.gov/. This is a flavone molecule with hydroxy groups attached at positions 4′, 5, and 7 [14]. Plants such as celery, parsley, chamomile, and various other culinary and medicinal plants produce it as a defense mechanism against detrimental environmental variables and pathogens, as well as a controller of metabolic processes [15]. Apigenin is typically found in plants as either a polymer or O- and C-glucosides. Although O-glucosides are more prevalent, C-glucosides are generally more easily absorbed. However, certain plants also contain apigenin in the form of aglycone, as well as its O-methyl ethers or acetylated derivatives [16]. Apigenin is mostly absorbed in the small intestine and undergoes significant metabolism through glucuronidation and sulfation mechanisms [17]. Following the ingestion of a plant containing apigenin, the apigenin glycoside is processed through recirculation, hydrolysis, and reduction to generate the monoglycoside. Additionally, it undergoes conjugation in the liver, intestines, or gut microbiota to produce a bioavailable glucuronide [17,18,19,20]. The prolonged retention of apigenin in the bloodstream and its gradual breakdown in the liver contribute to its increased bioavailability throughout the body [17]. Alternatively, apigenin can be readily integrated into nanoformulations either by itself or in conjunction with other active substances for therapeutic applications. To enhance the stability and bioavailability of apigenin for these objectives, it can be achieved by the creation of chemical analogs or by utilizing carriers such as emulsions, nanostructured lipid carriers, hydrogels, triterpenoid fridelin, or liposomes [18,21,22,23,24].

Apigenin is widely recognized for its significant physiological and therapeutic impacts on oxidative processes, viability, inflammation, and estrogen receptors in different organs [24,25]. The physiological and therapeutic effects of apigenin determine its appropriateness for treating many physiological diseases, such as cancer, nervous system disorders, cardiovascular conditions, diabetes, infectious diseases, hormonal issues, and other disorders [1,23,26]. In addition to having the capacity to reduce fat stores and the glucose metabolism, vitexin, which is an apigenin-8-C-glucoside, has been shown to possess antioxidative, anti-inflammatory, anticancer, neuroprotective, hepatoprotective, and cardioprotective qualities [27]. On the other hand, it is probable that these effects were not brought about by the apigenin-8-C-glucoside itself, but rather by its metabolite, apigenin, or by other metabolites. However, it is important to note that the majority of clinical studies conducted on apigenin have been affected by limited sample sizes, short trial durations, the absence of proper control groups (patients not receiving treatment), the utilization of apigenin from different sources and in varying doses (often exceeding normal physiological levels), supplements with varying aglycone contents, and other methodological weaknesses. Thus, there is currently a lack of robust and scientifically valid evidence supporting the efficacy of apigenin in lowering the risks and symptoms of any disease [18,28,29]. The cytotoxicity of apigenin towards healthy cells is rather minimal. Apigenin has not been found to have any mutagenic effect. However, it is important to consider potential medication interactions with apigenin prior to its administration.

## 3. Potential Benefits of Apigenin for Female Reproductive Health

Apigenin has attracted attention due to its possible advantages for female reproductive health. Below, a few of the potential advantages of apigenin in female reproductive health are presented in each passage.

### 3.1. Apigenin Affects Ovarian and Reproductive States

Apigenin’s anticancer characteristics also contribute to the preservation of ovarian health. Ovarian cancer is a highly deadly form of cancer that affects the female reproductive system [30], and it is important to have procedures in place to avoid it. Apigenin has been discovered to trigger apoptosis and hinder the growth of ovarian cancer cells [31]. This is accomplished through many processes, such as the regulation of cell cycle regulators and the initiation of apoptotic pathways. Apigenin aids in the preservation of ovarian function and general reproductive health by inhibiting the growth and dissemination of cancer cells [32]. Furthermore, apigenin’s influence also encompasses the wider reproductive condition. It could boost fertility by enhancing the quality of oocytes (egg cells) and facilitating embryo implantation [33]. Apigenin supplementation in animal experiments has demonstrated an increase in successful pregnancies, suggesting its potential as a natural fertility booster [34]. Therefore, apigenin has been documented to alleviate the detrimental impacts of environmental pollutants and endocrine disruptors on reproductive health, offering an extra level of safeguarding for reproductive functions.

Apigenin has demonstrated the ability to improve the maturation of oocytes and the development of embryos, most likely due to its antioxidant and antiapoptotic properties [35]. Apigenin aids in preserving the integrity and viability of oocytes by minimizing oxidative damage and supporting cell survival pathways [36]. Apigenin could regulate the levels of progesterone as well as estrogen, which are both essential for the proper functioning of the menstrual cycle and for supporting pregnancy [37]. Apigenin can assist in regulating hormonal balance by interacting with estrogen receptors and modulating their signaling pathways, consequently supporting normal reproductive processes [38]. Apigenin increases the ability of cells to withstand cause damage by increasing the production of antioxidant enzymes and decreasing the production of proinflammatory substances in ovarian cells [11]. Despite the unclear chemical activity of apigenin, its effects on the female reproductive system are vital to reproductive health. Furthermore, apigenin regulates other signaling pathways, such as the previously stated PI3K/Akt pathway, the nuclear factor kappa-light-chain-enhancer of activated B cells (NF-κB) pathway, and the mitogen-activated protein kinase (MAPK) pathway [1,39]. These pathways play a crucial role in cell proliferation, apoptosis, and inflammation, which are all essential for reproductive health [40]. Apigenin optimizes these pathways to support cell viability, diminish inflammation, and augment tissue restoration and regeneration [41]. The therapeutic capacity of apigenin in the field of reproductive biology and medicine is extensive. The potential of this treatment to boost ovarian function, improve the quality of oocytes, control hormonal balance, and provide protection against environmental stresses makes it a highly attractive option for addressing many reproductive diseases [42]. Apigenin has the potential to serve as a supplementary treatment in clinical settings to enhance fertility therapies, regulate disorders such as PCOS and POF, and enhance general reproductive well-being [43]. Apigenin primarily influences the ovaries by regulating oxidative stress [44]. The ovaries are very prone to oxidative injury because of their elevated metabolic activity and the production of ROS.

### 3.2. Apigenin Affects Ovarian and Uterine Cell Functions

**Figure 2:**
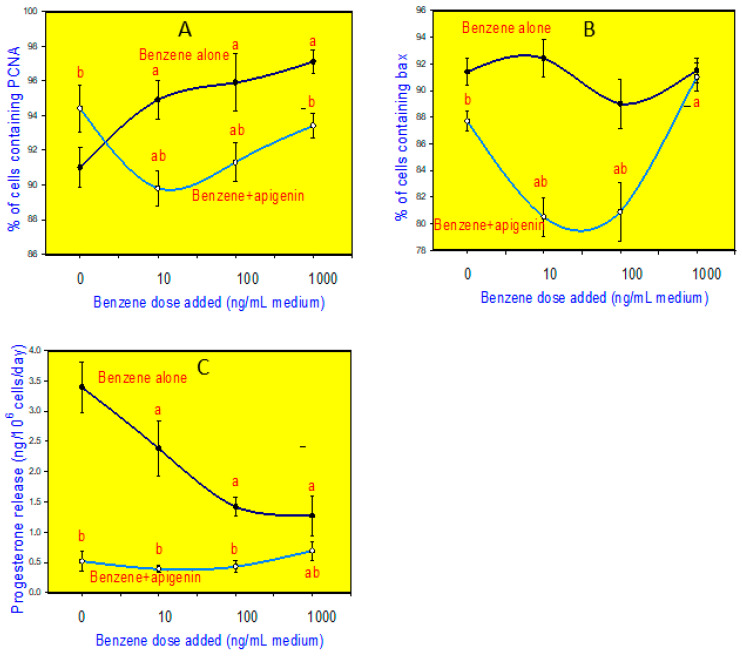
The addition of apigenin can promote proliferation (the accumulation of proliferating cell nuclear antigen, CNA) (**A**) to suppress apoptosis (the accumulation of Bax) (**B**) and progesterone release (**C**), as well as to prevent and invert the effect of benzene on these parameters, in cultured porcine granulosa cells [47]. a—significant effect of benzene: significant (*p* < 0.05) differences between the cells cultured without and with benzene; b—significant influence of apigenin on cells cultured with and without benzene: significant (*p* < 0.05) difference between the corresponding groups of cells cultured with or without apigenin.

Apigenin not only affects ovarian cells but also has an impact on uterine cell functioning, which demonstrates potential in the regulation of ovarian cell activities. The ovaries play a vital role in the generation of oocytes and the release of hormones such as estrogen and progesterone, which are necessary for reproductive well-being and the regulation of the menstrual cycle [45]. Studies have shown that apigenin has the ability to affect ovarian cancer cell lines by promoting apoptosis and suppressing cell growth [31]. Research has demonstrated that apigenin has the ability to decrease the production of antiapoptotic proteins, such as Bcl-2, while increasing the production of proapoptotic proteins, like Bax [46]. As a result, this promotes the death of ovarian cancer cells. Apigenin’s capacity to regulate apoptotic pathways indicates that it could be a powerful agent in decreasing the viability of malignant ovarian cells. In some in vitro experiments, the addition of apigenin to cultured porcine ovarian granulosa cells promoted their viability and proliferation (the accumulation of PCNA) (Figure 2A), and reduced the accumulation of the apoptosis marker Bax (Figure 2B), quantified by immunocytochemistry [47,48,49]. This effect, however, has not been observed in some similar experiments performed on the same model [50]. However, addition of apigenin to cultured porcine granulosa cells promoted estradiol and testosterone, and suppressed progesterone output (Figure 2C) [6].

**Figure 3:**
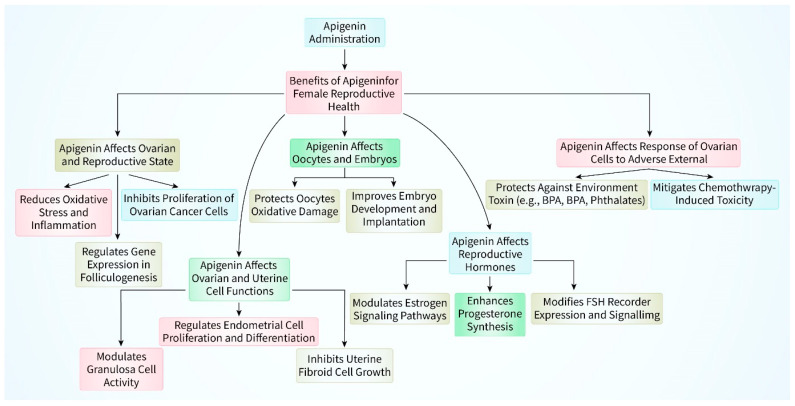
Apigenin could be beneficial to the reproductive health of women. The ovarian and reproductive status are both impacted by apigenin. Apigenin has an effect on the functioning of ovarian and uterine cells. Apigenin has an effect on both embryos and oocytes. The reproductive hormones are influenced by apigenin. The response of ovarian cells to harmful components from the environment is influenced by apigenin.

Moreover, apigenin has been reported to provide anti-inflammatory properties on ovarian cells [51]. Ovarian cancer is more likely to occur when there is long-term inflammation in the ovarian microenvironment. The suppressive effect of apigenin on the expression of proinflammatory cytokines such as IL-6 and TNF-α indicates that it could potentially alleviate ovarian diseases associated with inflammation [52]. Apigenin has been discovered to impact the growth and death of cells in the uterus, much like it does in ovarian cells [53]. Apigenin has the ability to cause cell cycle arrest, specifically at the G2/M phase, and initiate apoptosis in uterine cancer cell lines, indicating its potential as a promising anticancer drug [54]. Furthermore, the significance of apigenin in regulating estrogen receptor (ER) function is very remarkable. Apigenin has the ability to attach itself to estrogen receptors and regulate their function [55]. This can have the following two advantages: it can decrease the growth of cancer cells that rely on estrogen and it may also help relieve the symptoms of conditions caused by excessive estrogen such as endometriosis. Furthermore, the ability of apigenin to inhibit the formation of new blood vessels (anti-angiogenic qualities) plays a role in its possible therapeutic benefits for uterine health [56]. Apigenin can impede the process of angiogenesis, which results in a decrease in the blood flow to developing tumors. However, multiple investigations have shown that apigenin has the capacity to inhibit the structural abnormalities associated with ovarian cancer in the cell lines A2780, OVCAR-3, and SKOV-3 [57,58,59]. Apigenin, when applied to cultivated ovarian cancer cells, reduced cell viability, halted cell cycle progression at various stages, and stimulated cell death [60,61]. Apigenin has the ability to directly impact uterine cells. Apigenin demonstrated the ability to prevent contractions of isolated murine uterine tissue and to limit the growth of cultured uterine Ishikawa cells [62,63]. Therefore, apigenin’s anti-inflammatory capability, combined with its antioxidant benefits, makes it a promising candidate for safeguarding against ovarian malfunction and disorders (Figure 3).

### 3.3. Apigenin Affects Oocytes and Embryos

The quality of oocytes is a crucial factor in determining the success of fertilization and the growth of embryos. Research suggests that apigenin has a beneficial effect on the process of oocyte maturation [64]. Apigenin is thought to improve the maturation process by virtue of its antioxidant characteristics, which aid in reducing oxidative stress [53]. Oxidative stress has the potential to harm oocytes, leading to a decline in their capacity to undergo maturation and function optimally. Apigenin aids in preserving the cellular environment of the oocyte by minimizing oxidative damage [65]. Furthermore, the significance of apigenin in regulating intracellular signaling pathways is remarkable. It has been established that it interacts with important signaling molecules like cyclin-dependent kinases (CDKs) and mitogen-activated protein kinases (MAPKs), which play a critical role in regulating the cell cycle and maturation processes [51,66]. These interactions indicate that apigenin can facilitate the smooth advancement of oocytes through the stages of maturation, hence improving their ability to mature.

In addition to its impact on oocyte maturation, apigenin also influences the initial phases of embryonic development. Studies have shown that apigenin has the ability to protect embryos from oxidative stress, especially during the early stages of development when cells are proliferating and differentiating [67]. Apigenin aids in the promotion of embryonic growth by decreasing the levels of ROS and establishing a more stable environment [68]. Apigenin has also been discovered to impact gene expression associated with embryogenesis [69]. It regulates the activity of genes that are responsible for cell growth, specialization, and apoptosis [70]. This regulation ensures the homeostasis of cell survival and apoptosis, which is essential for proper embryogenesis.

### 3.4. Apigenin Affects Reproductive Hormones

Reproductive hormones such as estrogen, progesterone, testosterone, and luteinizing hormone (LH) are essential for controlling reproductive activities in both males and females. Any modifications in the amounts or functioning of these hormones can have significant impacts on reproductive health, fertility, and the overall state of health. In vivo studies demonstrated the ability of apigenin injections to decrease LH and to increase FSH levels in the plasma of rats with polycystic ovarian syndrome, and the injections increased the progesterone and decreased the testosterone and estradiol concentrations [4,71]. In contrast, the administration of a plant extract containing apigenin and apigenin glucosides resulted in an elevation of estradiol levels in the plasma of mice. This therapy also enhanced the expression of α-estrogen receptors in the uterus [15]. Apigenin effectively inhibited the secretion of insulin-like growth factor I (IGF-I) by cultivated porcine granulosa cells [49]. Apigenin treatment reduced the production of the inflammatory cytokines tumor necrosis factor-α (TNF-α) and interleukin-6 (IL-6) in the blood of rats with polycystic ovarian syndrome. However, it had no effect on IL-6 production in cultured human ovarian cancer cells [71,72]. Ultimately, apigenin reduced the generation of vascular endothelial growth factor (VEGF) by ovarian cancer cells [73,74].

The effect of apigenin on estrogen and progesterone is particularly intriguing in females [75]. Research has shown that apigenin has the ability to regulate the menstrual cycle by influencing the amounts of these hormones [76]. For instance, the addition of apigenin to animal models has resulted in changes in the estrous cycle, indicating its capacity to impact the regularity of menstruation and ovulation [53]. Furthermore, the anti-estrogenic characteristics of apigenin may provide therapeutic advantages in illnesses such as polycystic ovarian syndrome (PCOS) and estrogen-sensitive malignancies like breast cancer [77]. Apigenin has the potential to alleviate the symptoms related to high estrogen activity, such as irregular menstrual cycles, infertility, and the heightened risk of cancer, by lowering estrogen dominance [78].

### 3.5. Apigenin Affects Response of Ovarian Cells to Adverse External Factors

Apigenin exerts its influence on ovarian cells primarily through its antioxidant action. Apigenin aids in safeguarding ovarian cells from oxidative stress, a prevalent detrimental factor associated with diverse reproductive problems and ovarian malignancies, by scavenging free radicals and augmenting the body’s antioxidant defense mechanisms [79]. Oxidative stress has the potential to harm the various parts of cells, resulting in impaired cell function and eventual death. Apigenin’s capacity to counteract reactive oxygen species (ROS) aids in preserving cellular integrity and functionality [80]. Furthermore, studies have demonstrated that apigenin could regulate inflammatory pathways. Inflammation is a detrimental element that can have negative effects on ovarian cells, leading to the development of disorders like polycystic ovary syndrome (PCOS) and endometriosis [81,82]. Apigenin suppresses the synthesis of proinflammatory cytokines and enzymes, hence diminishing inflammation and its deleterious impact on ovarian cells [11]. The anti-inflammatory effect of this action aids in preserving the normal functioning of the ovaries and may perhaps decrease the likelihood of ovarian disorders associated with inflammation [39]. Apigenin demonstrates anticancer characteristics, which are especially pertinent to ovarian cells due to the elevated occurrence of ovarian cancer [83]. It triggers apoptosis, which is the programmed death of cancer cells, and prevents the growth and formation of blood vessels, and the spread of cancer cells [84]. Apigenin targets numerous pathways involved in cancer progression, which not only prevents the initiation of cancer but also controls its spread [83]. This improves the responsiveness of ovarian cells to oncogenic stimuli. To summarize, apigenin possesses multiple beneficial effects such as antioxidant, anti-inflammatory, and anticancer properties, which improve the ability of ovarian cells to withstand negative external influences. The significance of conducting additional research and development in this sector is highlighted by its potential as a therapeutic agent for safeguarding ovarian health and addressing ovarian disorders.

## 4. Molecular Mechanisms and Signaling Targets of Apigenin on Female Reproductive Organs

**Figure 4:**
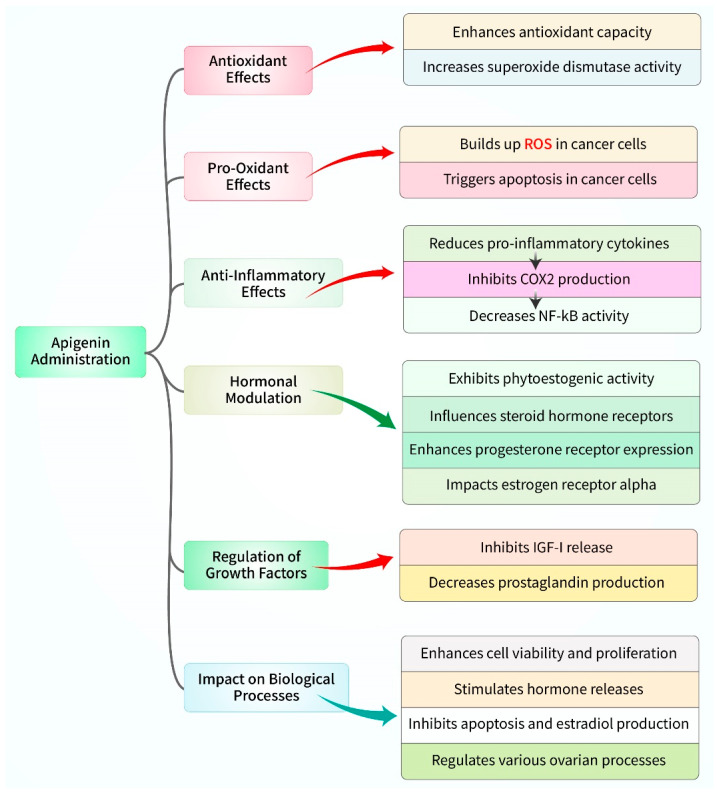
The influence of apigenin on female reproductive organs and its molecular mechanisms and signaling targets.

Multiple signaling pathways have been found to be involved in mediating the effects of apigenin on reproductive processes, as suggested by numerous in vitro and in vivo investigations, which are presented in Figure 4. Apigenin administration enhanced the overall antioxidant capacity and superoxide dismutase activity in rat plasma while reducing its oxidative status. It suggests that the favorable effects of apigenin on the hormonal status and ovarian functions of rats may be attributed to its antioxidant activity [71,85]. Contrarily, apigenin caused the buildup of reactive oxygen species in both embryonic fibroblasts and cultivated cancer cells, resulting in a decrease in their ability to survive [57,86]. The data suggest that apigenin has antioxidant effects on healthy ovarian cells in living organisms, while also having harmful pro-oxidant effects on cultured embryonal and cancer cells. Apigenin’s potential as an antioxidant on healthy gestational tissues should not be disregarded. Apigenin was able to suppress oxidative stress and resulted in apoptosis in cultured ovarian cells [82]. It decreased the amount of 8-isoprostane, which is a substance that indicates oxidative stress, in cultures of healthy placenta, fetal membranes, and myometrial cells [87]. Thus, apigenin has the ability to inhibit oxidative stress in normal reproductive tissues while promoting it in ovarian cancer cells. Apigenin, when added to ovarian cancer cell culture, decreased the intracellular expression of tyrosine kinase receptors, which are responsible for promoting cell viability and proliferation. Additionally, it maintained the anti-proliferative transcription factor p53 [73]. Unlike cancer cells, the introduction of apigenin to healthy porcine granulosa cells in culture resulted in an increase in BrdU incorporation (a marker of DNA synthesis). Furthermore, it promoted the accumulation of PCNA (a promoter of DNA polymerase and the S-phase of the cell cycle) and cyclin B1 (a promoter of the transition from the G- to M-phase of mitosis). This upregulation of cell cycle regulators was associated with enhanced cell viability [49]. Reactive oxygen species trigger cytoplasmic/intrinsic apoptosis, leading to the accumulation of the apoptosis marker and promoter Bax, as well as a decrease in mitochondrial membrane potential, in ovarian cancer cells. Apigenin analogs have been accountable for promoting this effect [88]. These data suggest that apigenin can limit the viability of cancer cells and stimulate the growth of healthy ovarian cells via affecting the regulators of the proliferation of cells [53].

An excess of ROS can result in ovarian dysfunction, which has a negative impact on both follicular development and oocyte quality. These factors are crucial for maintaining fertility. Apigenin, due to its robust antioxidant characteristics, aids in the neutralization of ROS, thereby safeguarding ovarian cells against oxidative harm [89]. This safeguard can enhance the overall well-being and operation of the ovaries, bolstering typical reproductive activities. Apigenin not only possesses antioxidant properties, but also affects important signaling pathways that are essential for ovarian function [51]. For example, it could regulate the expression of genes that are involved in steroidogenesis, which is the biological process responsible for producing steroid hormones [90]. Steroid hormones, including estrogen and progesterone, play a crucial role in regulating the menstrual cycle and sustaining pregnancy. Apigenin’s capacity to control these hormones indicates that it can assist in maintaining the hormonal equilibrium, a crucial factor for reproductive well-being [43]. Furthermore, chronic inflammation has been observed to affect ovarian function and play a role in certain reproductive illnesses, such as polycystic ovary syndrome (PCOS) and endometriosis. Research has shown that apigenin could inhibit the creation of proinflammatory cytokines and restrain the activation of inflammatory pathways, resulting in a decrease in inflammation in ovarian tissues [67].

Apigenin injections reduced the levels of inflammatory markers in the blood of rats with experimentally generated polycystic ovarian syndrome. Hence, apigenin has the ability to inhibit inflammatory processes linked to polycystic ovarian syndrome and other reproductive dysfunctions [71]. The suppressive effect of apigenin on ovarian cancer can be attributed to its anti-inflammatory function. This idea is supported by the ability of apigenin to reduce the levels of the proinflammatory cytokines TNF-α and IL-6, as well as COX2, in the plasma of rats [73,91]. In cultured human placenta, fetal membranes, and myometrial cells, the addition of apigenin decreased the production of the proinflammatory transcription factor NF-κB, the release and gene expression of the cytokines IL-6 and IL-8, as well as COX2, the subsequent release of the prostaglandins PGE2 and PGF2α, and the expression and activity of the matrix-degrading enzyme matrix metalloproteinase 9 (MMP-9) [73]. Hence, most of the conducted research has shown that apigenin has an anti-inflammatory effect on both healthy and cancerous ovarian cells. This effect is achieved by reducing the activity of many factors involved in promoting inflammation both inside and outside of the cells. Apigenin’s capacity to inhibit inflammation suggests its potential in mitigating ovarian illnesses such as cancer and polycystic ovarian syndrome. Hence, the impact of apigenin on different reproductive tissues may be attributed to its anti-inflammatory properties.

Apigenin has also demonstrated its anti-inflammatory effects on healthy gestational tissues. Apigenin decreased the expression of the proinflammatory transcription factors NF-κB and Nrf2 in the endometrium of rats under in vivo circumstances [92]. Moreover, it blocked the attachment of the proinflammatory transcription factor NF-κB to DNA, the release and expression of genes, the cytokines IL-6 and IL-8, as well as COX2, and the subsequent release of the prostaglandins PGE2 and PGF2α, as well as the expression and activity of the matrix-degrading enzyme matrix metalloproteinase 9 (MMP-9) in cultured human placenta, fetal membranes, and myometrial cells [87]. Apigenin effectively decreased the concentration of nitric oxide, a relaxing component derived from the endothelium, within the ovaries of rats. Additionally, it successfully suppressed the generation of VEGF by cancer cells cultivated in a laboratory setting [93,94]. These compounds are recognized as stimulators of angiogenesis. These data suggest that apigenin may impact ovarian functioning by altering ovarian angiogenesis and blood flow. The impact of apigenin on ovarian steroid hormones has been extensively studied. Steroid hormones, through their receptors, induce ovarian folliculogenesis, oogenesis, gestational tissues, and many reproductive diseases [63,95,96,97]. Apigenin exhibits phytoestrogenic activity, resembling that of endogenous steroid hormones. This activity can potentially impact the generation of steroid hormones through both direct and feedback mechanisms [98,99,100,101]. Apigenin’s capacity to influence steroid hormones suggests that it may impact female reproductive processes by altering the release of these hormones.

Moreover, it is possible to suggest that the phytoestrogen apigenin directly affects the receptors of steroid hormones. Apigenin enhances the expression of nuclear progesterone receptors in cultured uterine Ishikawa cells [63,102]. Apigenin glucosides stimulated the growth of the uterus in mice and increased the expression of estrogen receptor alpha in the uteri of mice. However, the influence of apigenin on cultured uterine cells in mice was inhibited by an antagonist of estrogen receptor alpha but not estrogen receptor beta [101]. The results indicate that apigenin is a phytosteroid that can impact the female reproductive system, specifically the uterus, via activating steroid hormone receptors and the subsequent intracellular pathway. The observations suggest that steroid hormones and their receptors may play a significant role in mediating the effects of apigenin on female reproductive processes that are dependent on steroids.

The presence of apigenin in cultured healthy porcine ovarian granulosa cells has been found to inhibit the release of IGF-I. This suggests that apigenin has the ability to affect ovarian functions through its influence on IGF-I. IGF-I plays a crucial role in regulating ovarian steroidogenesis, ovarian cell proliferation and viability, ovarian folliculogenesis, as well as suppressing ovarian cell apoptosis and the development of ovarian cancer [49,95,103]. Moreover, prostaglandins play a crucial role in regulating both ovarian and uterine activities [95]. Apigenin has the ability to decrease the production of COX2, which is an important enzyme involved in the manufacturing of prostaglandins in ovarian cancer cells and in the myometrium. Additionally, it can also inhibit the release of the prostaglandins PGE2 and PGF2α by the cells of the myometrium [87]. These data indicate that prostaglandins may serve as the next endocrine mediator of apigenin’s effects on the ovary and uterine. This, in turn, enhanced the viability and proliferation of cultured porcine ovarian granulosa cells. Additionally, it stimulated the release of progesterone, IGF-I, oxytocin, and PGE 2, while inhibiting apoptosis and estradiol production [49]. Moreover, steroid hormones and IGF-I serve as established regulators of various processes in the ovaries, including cell proliferation, apoptosis, follicle development, egg production, pregnancy, and embryo formation [95]. Thus, the impact of apigenin on steroid hormones, IGF-I, and their receptors can facilitate the effects of apigenin on various biological processes.

**Figure 5:**
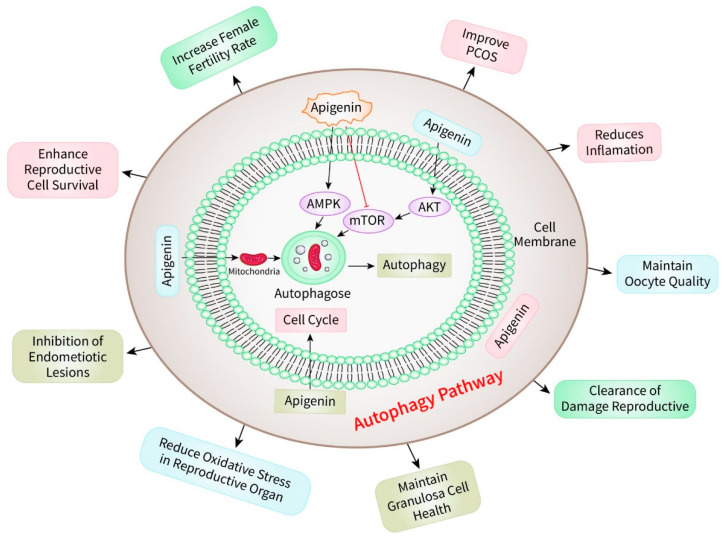
The involvement of autophagy in the regulation of reproductive function by apigenin.

Autophagy is a process of breaking down substances in the body that is controlled by specific proteins and signaling pathways, such as the mammalian target of rapamycin (mTOR) and the adenosine monophosphate-activated protein kinase (AMPK) [104,105]. Autophagy plays a crucial role in the functioning of different reproductive cells, such as oocytes (egg cells), sperm, and supportive somatic cells in the reproductive system, within the framework of reproductive health [106]. The process of autophagy is one of the ways that apigenin uses to exert its impacts on reproductive health [107]. It has been demonstrated that apigenin could influence autophagy, which, in turn, affects reproductive function [108]. Apigenin, for example, has been demonstrated to activate autophagy in ovarian cells by blocking the mTOR pathway, which is a negative regulator of autophagy [109]. Additionally, the elimination of damaged mitochondria and other organelles can be facilitated by this autophagic activity, which ultimately results in an improvement in the quality and functionality of oocytes [110]. Apigenin’s significance in autophagy extends to its effects on granulosa cells, which are essential for the maturation and development of oocytes [12]. Apigenin-induced autophagy in granulosa cells has the potential to increase cellular survival under stressful situations such as oxidative stress, which is known to decrease reproductive function [111]. Apigenin serves to preserve the health and viability of granulosa cells by increasing the autophagic flow [112]. This, in turn, benefits the maturation and quality of oocytes in a roundabout way. The role that autophagy plays in the reproductive function that is mediated by apigenin is demonstrated in Figure 5.

Additionally, the antioxidant properties of apigenin are closely associated with its ability to induce autophagy [113]. Apigenin lowers the amount of damage that is caused to reproductive cells and tissues by lowering the amount of oxidative stress [114], which further enhances reproductive function. Apigenin is a powerful mechanism that protects and improves reproductive health. It provides protection and improvement through its dual action of antioxidation and autophagy induction [11]. Through the process of inducing autophagy, apigenin can play a crucial role in the regulation of reproductive function [11]. Apigenin can improve the quality of reproductive cells, enhance cell survival, and promote the clearance of damaged cellular components [115]. This multidimensional approach not only allows for the maturation and function of oocytes and sperm, but also ensures that the reproductive system is in good health. It is becoming increasingly clear that apigenin has the potential to be used as a medicinal agent in the field of reproductive health as research continues to unearth the precise aspects of its actions.

## 5. Therapeutic Application of Apigenin in Reproductive Biology and Medicine

The ability of apigenin to prevent oxidative stress, to affect estrogen receptors, the release of reproductive hormones, and the functions of ovarian and uterine cells, and to promote ovarian folliculogenesis, oogenesis, and embryogenesis, described above, indicates its potential applicability for the increase in fecundity in assisted reproduction and animal production. It is not to be excluded that the addition of apigenin to culture medium can promote the in vitro maturation and fertilization of oocytes, and the dietary addition of apigenin could increase the efficiency and fecundity of these processes in vivo. On the other hand, the available literature does not contain reports concerning clinical trials of apigenin application. Therefore, the applicability of apigenin in assisted reproduction and animal production requires further elucidation.

**Figure 6:**
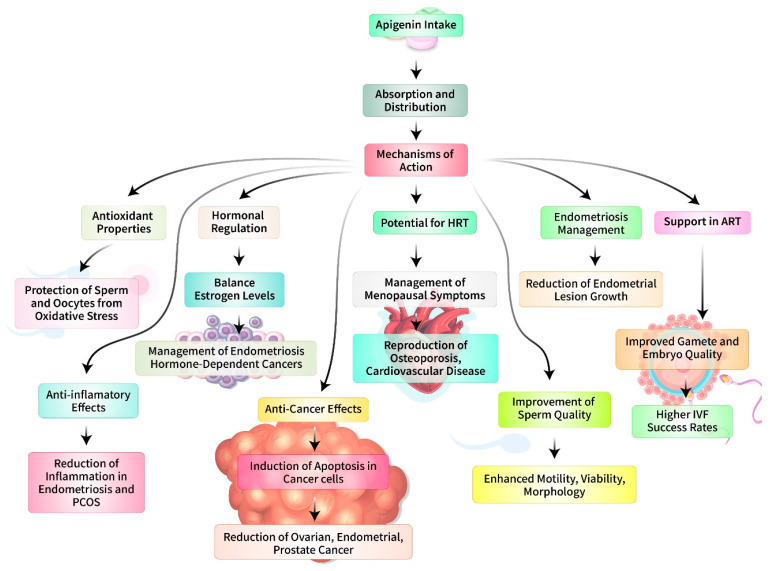
The use of apigenin in the field of reproductive biology and medicine for therapeutic purposes.

The anti-inflammatory, antioxidant, and anticancer characteristics of apigenin offer exciting new directions in reproductive biology and medicine for the management of a range of reproductive disorders. Inflammation and oxidative stress are prevalent underlying variables in various reproductive diseases such as polycystic ovarian syndrome (PCOS), endometriosis, and infertility [116]. Apigenin possesses strong anti-inflammatory and antioxidant properties that can alleviate these diseases by regulating inflammatory pathways and minimizing the damage caused by oxidative stress [117]. Apigenin has demonstrated the ability to reduce oxidative stress and suppress the activity of inflammatory cytokines, including TNF-α, IL-6, and IL-1β, which are found at higher levels in individuals with PCOS [118]. Apigenin has the potential to restore normal ovarian function and enhance reproductive outcomes in women with PCOS by mitigating these variables [119]. Apigenin has the capacity to decrease the activity of proinflammatory cytokines and boost the body’s antioxidant defenses [120]. This can help alleviate symptoms and slow down the development of endometriotic lesions. Research has shown that apigenin could decrease the survival and invasive properties of endometrial cells, indicating its potential as a therapeutic agent for treating endometriosis [121]. Apigenin has demonstrated significant anticancer efficacy in multiple cancer types, including reproductive system malignancies like ovarian and cervical tumors [83]. The processes by which it works include triggering apoptosis, decreasing cell growth, and suppressing angiogenesis [122]. Figure 6 illustrates the therapeutic use of apigenin in the field of reproductive biology and medicine.

Apigenin demonstrates anticancer properties by triggering apoptosis through the intrinsic mitochondrial pathway, blocking crucial signaling pathways like PI3K/Akt, and lowering angiogenesis by reducing the expression of vascular endothelial growth factor (VEGF) [123]. These interventions have the potential to decrease the growth of tumors and their spread to other parts of the body, thereby boosting the effectiveness of current therapies and potentially increasing the chances of survival [124]. Apigenin, with its therapeutic qualities, can be beneficial in the treatment of cervical cancer, which is primarily caused by persistent infection with high-risk human papillomavirus (HPV) [125]. Apigenin has been discovered to downregulate the expression of the HPV16 and HPV18 oncoproteins, which play a critical role in the development of cervical cancer [126]. Moreover, it could trigger cell cycle arrest and death in cervical cancer cells [127]. The coadministration of apigenin with traditional chemotherapeutic drugs has the potential to augment treatment effectiveness and mitigate negative side effects [128]. Apigenin plays a significant function in improving fertility by positively impacting the reproductive health of both males and females [129]. Apigenin has demonstrated efficacy in increasing sperm quality and function in males by mitigating oxidative stress and augmenting antioxidant defenses [130]. Apigenin could promote ovarian function and provide protection against ovarian toxicity caused by environmental pollutants or chemotherapy in women [131]. Apigenin’s antioxidant qualities can safeguard spermatozoa from oxidative harm, therefore enhancing sperm parameters [64]. Research has shown that taking apigenin supplements can enhance the number, movement, and survival of sperm, suggesting that it could be a viable treatment for male infertility [64]. Apigenin could improve ovarian function and provide protection against symptoms of PCOS in the context of female reproductive health [4]. Chemotherapy-induced ovarian toxicity is a major worry for young cancer patients, since it might result in premature ovarian failure and infertility. Apigenin’s antioxidant properties and ability to prevent programmed cell death can help maintain the number and function of eggs in the ovaries during and after chemotherapy [1]. Furthermore, its capacity to regulate the hormonal equilibrium and promote the development of follicles can improve reproductive results. The use of apigenin as an adjuvant to assisted reproductive technologies (ARTs), such as in vitro fertilization (IVF), can enhance outcomes [132]. Oxidative stress and inflammation have negative effects on the quality of oocytes, the development of embryos, and the success of implantation [133]. The antioxidative and anti-inflammatory characteristics of apigenin can enhance the conditions for ART operations [134]. Apigenin’s power to decrease oxidative stress and promote antioxidant capability can enhance the quality of oocytes, which is vital for successful fertilization and embryo development [135]. Enhanced oocyte quality results in increased rates of embryo implantation and successful pregnancy in ART cycles [136]. Apigenin’s anti-inflammatory properties can improve the ability of the endometrium to accept an embryo, increasing the chances of successful implantation and decreasing the likelihood of early pregnancy termination [137]. Apigenin facilitates embryo implantation and growth by regulating the immune response and lowering uterine inflammation [138]. Apigenin is generally well-tolerated, exhibiting low levels of toxicity in both animal and human investigations [4]. Nevertheless, additional clinical trials are required to determine the most effective dosages, long-term safety, and effectiveness in different reproductive health situations.

## 6. Limitations and Future Perspectives of Apigenin in Reproductive Function and Medicine

To fully exploit its potential, it is necessary to address various limitations and topics for future research, despite the promising biological activity it possesses. A major drawback of apigenin is its limited ability to be absorbed and utilized by the body [17]. Upon ingestion, apigenin undergoes substantial metabolism in the liver and intestines, leading to a large decrease in its concentration in the bloodstream and, thus, its effectiveness [23]. Exploring strategies to enhance its bioavailability, such as employing nanoparticle carriers or coadministering it with metabolism-inhibiting agents, is crucial but still insufficiently investigated. Another issue lies in determining the best dosage of apigenin for medicinal reasons. Administering the large doses necessary to produce substantial physiological effects in animal models may be impractical or risky for human subjects. Comprehensive toxicity studies are necessary to determine the appropriate dosage levels that are safe. Moreover, there is a lack of comprehensive documentation regarding the lasting impacts of consuming apigenin, which highlights the need for additional research to ascertain its safety for prolonged use. Although apigenin has demonstrated many biological impacts, the specific mechanisms responsible for these activities remain incompletely known. Apigenin has a significant impact on various aspects of reproductive health, including hormone control, oxidative stress, and inflammation [53]. Gaining a more profound comprehension of the molecular targets and pathways is essential to further the development of targeted medicines. The effects of apigenin in reproductive medicine can be influenced by factors such as sex, the hormone condition, and the existence of reproductive diseases. For example, certain research proposes that apigenin could potentially improve fertility by enhancing the quality of sperm or regulating menstrual cycles [64]. However, other studies show possible negative consequences, such as interfering with endocrine processes. The contradictory findings highlight the necessity for further meticulous and situation-specific research to clearly define the advantages and drawbacks.

To address the problem of low bioavailability, it is crucial for future studies to concentrate on the development of sophisticated delivery systems. Nanotechnology, liposomal encapsulation, and the utilization of bio-enhancers hold great potential in augmenting the absorption and efficacy of apigenin [139]. These advancements have the potential to facilitate more effective therapeutic uses. Considering the diverse range of reactions to apigenin, the implementation of tailored treatment strategies could prove advantageous. Further research is required to investigate the genetic, hormonal, and lifestyle aspects that impact individual reactions to apigenin. Customizing apigenin-based therapies according to individual profiles could optimize the effectiveness and minimize the negative consequences. There is an urgent requirement for meticulously planned clinical trials to authenticate the effectiveness and safety of apigenin in the field of reproductive medicine. The trials should specifically target a range of reproductive disorders, including PCOS, endometriosis, and male infertility, to gather strong and reliable data for its therapeutic application. Furthermore, conducting long-term studies is crucial to comprehend the enduring effects of apigenin supplementation. Future studies should focus on uncovering the precise molecular processes through which apigenin influences reproductive health. This entails the identification of the precise genes, proteins, and signaling pathways that are involved in its mechanism of action. Acquiring such knowledge could enhance the progress of creating more accurate and efficient interventions using apigenin. Furthermore, exploring the combined effects of apigenin with other chemicals, such as other flavonoids, vitamins, or conventional pharmaceuticals, may amplify its therapeutic capacity. Gaining insight into the mechanisms by which apigenin interacts with other drugs has the potential to enhance the efficacy of combination therapies compared to treatments using a single agent.

## 7. Conclusions

**Figure 7:**
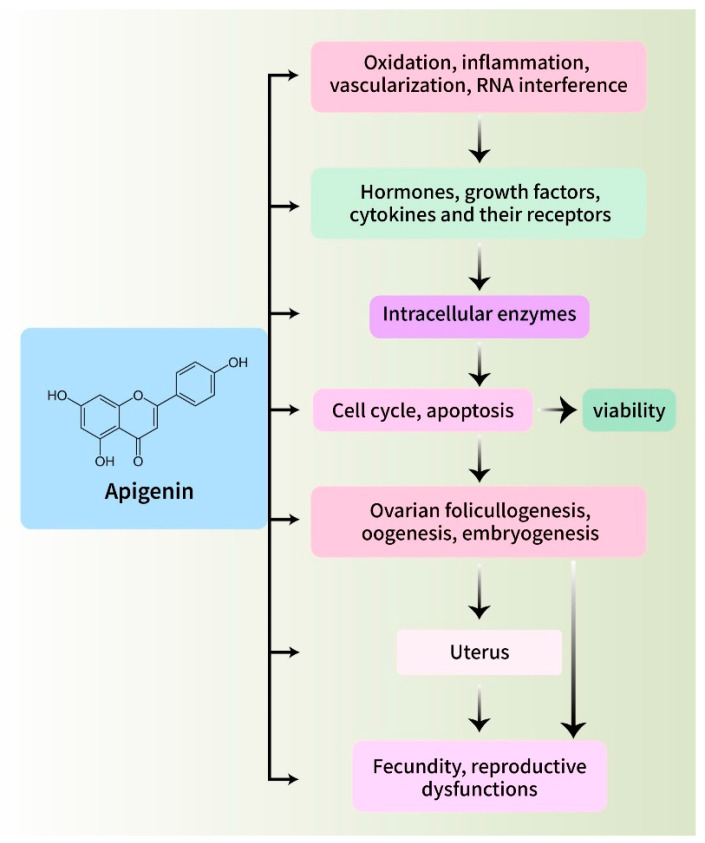
The most important functions that apigenin serves in the female reproductive system, as well as the linkages between those functions.

In the present study, we summarized the existing studies on the impact of apigenin on female reproductive processes and associated abnormalities. According to the existing scientific literature, apigenin has been found to enhance the development of ovarian follicles, boost the growth and survival of ovarian and embryonic cells, improve fertility, and stimulate the production of reproductive hormones through several methods [140]. Figure 7 illustrates the primary objectives of apigenin in the female reproductive system and their functional connections. However, apigenin could inhibit the activities of ovarian cancer cells and reduce the negative changes in the female reproductive system caused by environmental pollutants, harmful medications, cancer, polycystic ovarian syndrome, ischemia, and endometriosis [114,131]. The effects of apigenin on both normal and impaired reproductive processes may be attributed to its influence on oxidative processes, cellular proliferation, apoptosis, renewal and viability, ovarian blood circulation, hormone release and reception, and RNA interference [11]. The observed effects of apigenin suggest that it has the potential to enhance female reproductive processes and serve as a therapeutic agent for certain reproductive problems. Overall, apigenin is a powerful natural molecule that offers substantial advantages for the health of the female reproductive system [141]. Due to its wide range of biological activities and strong mechanisms of action, it is a great asset in the field of reproductive medicine. Apigenin has the potential to become a fundamental component in improving and treating female reproductive function as further research reveals its complete capabilities. Furthermore, apigenin’s importance in reproductive medicine is further highlighted by its capacity to increase fertility and improve ART outcomes. To fully realize apigenin’s therapeutic potential and develop it into successful treatments for diseases related to reproductive health, more studies and clinical trials will be required.

## Supplementary Materials

The following supporting information can be downloaded at: https://www.mdpi.com/article/10.3390/biomedicines12102405/s1, Table S1: Summary of reports concerning the effects of apigenin on processes related to female reproduction.

## Abbreviations

Bax—Bcl-2-associated X; Bcl-2—B-cell lymphoma 2; COX2—cyclooxygenase 2; FSH—follicle-stimulating hormone; IGF-I—insulin-like growth factor I; IL-6—interleukin-6; LH—luteinizing hormone; MMP-9—matrix metalloproteinase-9; NF-κB—nuclear factor kappa B; Nrf2—nuclear factor erythroid 2-related factor 2; PCNA—proliferating cell nuclear antigen; PGE2—prostaglandin E2; PGF2α—prostaglandin F2 α; RNA—ribonucleic acid; TNF-α—tumor necrosis factor-α; VEGF—vascular endothelial growth factor.
