# Supplementary material for: Apigenin as a Promising Agent for Enhancing Female Reproductive Function and Treating Associated Disorders
Source: Biomedicines. 2024 Oct 21;12(10):2405. doi: 10.3390/biomedicines12102405 (PMC11504338; doi:10.3390/biomedicines12102405)
Supplement: Supplementary file 1 [file biomedicines-12-02405-s001.zip › biomedicines-3198625-supplementary.pdf]

Table S1. Summary of reports concerning effects of apigenin on processes related to female reproduction.

Reproductive Process	Species	Effect	References
Ovarian folliculogenesis	Rat	Stimulation	Soyman et al., 2017; Darabi et al., 2020; Talebi et al., 2020
Ovarian cell proliferation	Pig	Stimulation	Sirotkin et al., 2020a,b; Fabova et al., 2023
		No effect	Sirotkin et al., 2021
	Human cancer cells	Inhibition	Chen et al., 2012; Suh et al., 2015; Tang et al., 2015; Pal et al., 2017; Ittiudomrak et al., 2019; Tavsan et al., 2019: Abd Ghani et al., 2020
Ovarian cell apoptosis	Rat	Inhibition	Telebi et al., 2020
	Pig	Inhibition	Sirotkin et al., 2020b; Fabova et al., 2023
	Pig	No effect	Sirotkin et al., 2021
	Human cancer cells	Stimulation	Chen et al., 2012; Suh et al., 2015; Pal et al., 2017; Ittiudomrak et al., 2019; Tavsan et al., 2019: Abd Ghani et al., 2020
Uterine cell functions	Mice	Inhibition	Dean et al., 2018; Suhas et al., 2018
	Mice	Stimulation	Yao et al., 2021; Yuan et al., 2023
	Rat	No effect	Barlas et al., 2014
Oogenesis	Mice	Stimulation	Talebi et al.,2020
	Goat	Stimulation	Silva et al., 2018
Embryogenesis	Mice	Stimulation	Safari et al., 2018
		Inhibition	Matsuo et al., 2005
Release of FSH by pituitary	Rat	Stimulation	Darabi et al., 2020
Release of LH by pituitary	Rat	Inhibition	Darabi et al., 2020
Release of progesterone by ovarian cells	Rat	Stimulation	Darabi et al., 2020
	Pig	Inhibition	Sirotkin et al.,2020a,b
		No effect	Sirotkin et al., 2021
Release of testosterone by ovarian cells	Rat	Inhibition	Darabi et al., 2020
	Pig	Stimulation	Sirotkin et al.,2020a,b
	Pig	No effect	Sirotkin et al., 2021
Release of estradiol by ovarian cells	Rat	Inhibition	Darabi et al., 2020
	Pig	Stimulation	Sirotkin et al.,2020a,b, 2021; Fabova et al., 2023
	Mice	Stimulation	Yuan et al., 2023
	Rat	Inhibition	Darabi et al., 2020

Reproductive Process	Species	Effect	References
Release of inflammatory cytokines by ovarian cells	Human cancer cells	No effect	Suh et al., 2015
Release of anti-Mullerian hormone by ovary	Rat	Stimulation	Talebi et al., 2020
		No effect	Soyman et al., 2017
Release of vascular endothelial growth factor (VEGF) by ovarian cells	Human cancer cells	Inhibition	Chen et al., 2012
Expression of miRNA miR-152 in ovarian cells	Pig	Stimulation	Fabova et al., 2023
Expression of progesterone receptor in uterine cells	Mice	Stimulation	Dean et al., 2018
Expression of estrogen α receptors in uterus	Mice	Stimulation	Yuan et al., 2023
Ovarian cancer	Human, rat	Inhibition	Chen et al., 2012; Suh et al., 2015; Tang et al., 2015; Pal et al., 2017; Ittiudomrak et al., 2019; Tavsan and Kavali, 2019; Tavsan et al., 2019: Abd Ghani et al., 2020; Talebi et al., 2020
Polycystic ovarian syndrome	Rat	Inhibition	Darabi et al., 2020
Ovarian ishemia	Rat	Inhibition	Soyman et al., 2017
Endometriosis	Rat	Inhibition	Jiang et al., 2018; Maresman et al., 2021
Response to adverse effects of chemotherapy	Rat	Inhibition	Talebi et al., 2020
	Human cancer cells	Stimulation	Pal et al., 2017
Response to adverse effects of ishemia	Rat	Inhibition	Soyman et al., 2017
Response to adverse effect of H_2O_2 and actinomycin	Rat	Inhibition	Safari et al., 2018
Response to benzene	Pig	Inhibition	Sirotkin, 2020b
Response to toluene	Pig	Inhibition	Sirotkin et al., 2021
Response to Cu nanoparticles	Pig	Inhibition	Sirotkin et al., 2020a
